# Case Report of Nephrogenic Diabetes Insipidus with a Novel Mutation in the *AQP2* Gene

**DOI:** 10.3390/ijms26157415

**Published:** 2025-08-01

**Authors:** Alejandro Padilla-Guzmán, Vanessa Amparo Ochoa-Jiménez, Jessica María Forero-Delgadillo, Karen Apraez-Murillo, Harry Pachajoa, Jaime M. Restrepo

**Affiliations:** 1 Centro de Investigaciones Clínicas, Fundación Valle del Lili, Cali 760032, Colombia; 2 Health Sciences Faculty, Universidad Icesi, Cali 760031, Colombiarestrepojaime@hotmail.com (J.M.R.); 3 Department of Maternal and Child Health, Pediatric Nephrology Service, Fundación Valle del Lili, Cali 760032, Colombia; 4 Medical Genetics Service, Fundación Valle del Lili, Cali 760032, Colombia

**Keywords:** nephrogenic diabetes insipidus, *AQP2*, polyuria, polydipsia, central diabetes insipidus

## Abstract

Nephrogenic diabetes insipidus (NDI) is a rare hereditary disorder characterized by renal resistance to arginine vasopressin (AVP), resulting in the kidneys’ inability to concentrate urine. Approximately 90% of NDI cases follow an X-linked inheritance pattern and are associated with pathogenic variants in the *AVPR2* gene, which encodes the vasopressin receptor type 2. The remaining 10% are attributed to mutations in the *AQP2* gene, which encodes aquaporin-2, and may follow either autosomal dominant or recessive inheritance patterns. We present the case of a male infant, younger than nine months of age, who was clinically diagnosed with NDI at six months. The patient presented recurrent episodes of polydipsia, polyuria, dehydration, hypernatremia, and persistently low urine osmolality. Despite adjustments in pharmacologic treatment and strict monitoring of urinary output, the clinical response remained suboptimal. Given the lack of improvement and the radiological finding of an absent posterior pituitary (neurohypophysis), the possibility of coexistent central diabetes insipidus (CDI) was raised, prompting a therapeutic trial with desmopressin. Nevertheless, in the absence of clinical improvement, desmopressin was discontinued. The patient’s management was continued with hydrochlorothiazide, ibuprofen, and a high-calorie diet restricted in sodium and protein, resulting in progressive clinical stabilization. Whole-exome sequencing identified a novel homozygous missense variant in the *AQP2* gene (c.398T > A; p.Val133Glu), classified as likely pathogenic according to the American College of Medical Genetics and Genomics (ACMG) criteria: PM2 (absent from population databases), PP2 (missense variant in a gene with a low rate of benign missense variation), and PP3 (multiple lines of computational evidence supporting a deleterious effect)]. NDI is typically diagnosed during early infancy due to the early onset of symptoms and the potential for severe complications if left untreated. In this case, although initial clinical suspicion included concomitant CDI, the timely initiation of supportive management and the subsequent incorporation of molecular diagnostics facilitated a definitive diagnosis. The identification of a previously unreported homozygous variant in *AQP2* contributed to diagnostic confirmation and therapeutic decision-making. The diagnosis and comprehensive management of NDI within the context of polyuria-polydipsia syndrome necessitates a multidisciplinary approach, integrating clinical evaluation with advanced molecular diagnostics. The novel *AQP2* c.398T > A (p.Val133Glu) variant described herein was associated with early and severe clinical manifestations, underscoring the importance of genetic testing in atypical or treatment-refractory presentations of diabetes insipidus.

## 1. Introduction

Nephrogenic diabetes insipidus (NDI) is a disorder characterized by the physiological inability to concentrate urine due to the kidneys’ inadequate response to antidiuretic hormone (ADH), also known as arginine vasopressin (AVP) [1,2,3]. The primary clinical manifestations of this condition include polydipsia, polyuria, and hypernatremia [2,3].

From a pathophysiological perspective, the disease involves impaired renal sensitivity to AVP, a hormone synthesized in the paraventricular and supraoptic nuclei of the hypothalamus and subsequently secreted by the neurohypophysis into the bloodstream in response to increased plasma osmolality (>290 mOsm/Kg) or hypovolemia. Under normal circumstances, plasma osmolality values exceeding this threshold induce a progressive increase in AVP secretion. This hormonal activation promotes phosphorylation, intracellular trafficking, and translocation of the aquaporin-2 (AQP2) water channel, facilitating its insertion into the apical membrane of collecting duct cells. As a result, water enters the cell and, subsequently, the vascular compartment, allowing for tubular water reabsorption and urinary concentration by the nephron [2,4].

Because NDI results from renal resistance to AVP, affected individuals excrete large volumes of dilute urine, leading to compensatory polydipsia. NDI exists in both primary and secondary forms. Primary forms, which are hereditary in origin, are caused by pathogenic variants in the *AVPR2* and *AQP2* genes [5].

In hereditary NDI, the estimated prevalence is approximately 1 in 5 million males. This condition is less common than central diabetes insipidus (CDI). X-linked forms, caused by mutations in *AVPR2*, account for roughly 90% of cases. The remaining 10% are associated with autosomal dominant or recessive inheritance patterns involving mutations in the *AQP2* gene [2,4,6,7,8].

We report the case of a male infant with a clinical diagnosis of NDI, in whom concomitant CDI was initially suspected due to the absence of the posterior pituitary bright spot on sella turcica magnetic resonance imaging. However, the lack of clinical response to desmopressin administration led to the exclusion of this hypothesis. The diagnosis was ultimately confirmed through whole-exome sequencing, which revealed a novel, homozygous, likely pathogenic, missense variant in exon 2 of the *AQP2* gene (c.398T > A; p.Val133Glu).

## 2. Case Description

A 9-month-old male infant from the Colombian southwest, born at term from non-consanguineous parents and previously healthy, was initially suspected of having NDI at six months of age. He was referred from a hospital in the Colombian southwest due to a clinical history of suspected cow’s milk protein allergy and gastroesophageal reflux disease since three months of age. He had been under follow-up by pediatric gastroenterology but developed significant weight loss, failure to thrive, delayed neurodevelopment, and severe protein–calorie malnutrition, prompting referral to the emergency department.

Upon admission, the patient was in fair general condition. Physical examination revealed generalized pallor, polyuria, severe dehydration, and a systolic murmur on cardiac auscultation. Initial laboratory findings demonstrated normal glucose and transaminase levels, venous blood gas analysis consistent with compensated metabolic acidosis, and a normal transthoracic echocardiogram. Additional investigations revealed hyposthenuria, hyperchloremia, hypernatremia, decreased urine osmolality (see Table 1), the absence of proteinuria, and an estimated glomerular filtration rate (eGFR) of 87 mL/min/1.73 m^2^ based on the modified Schwartz formula [9].

Due to the clinical suspicion of CDI, a therapeutic trial of desmopressin was performed on two separate occasions, both without clinical response. Based on this lack of response, a diagnosis of NDI was established, and treatment was initiated with celecoxib and hydrochlorothiazide, resulting in a transient and variable reduction in urine output and serum sodium levels. Subsequently, the patient’s condition worsened due to an episode of sepsis, likely of respiratory and urinary origin, and antibiotic therapy was initiated.

Considering persistent polyuria and low urine osmolality, a diagnosis of mixed diabetes insipidus (central and nephrogenic) was proposed, prompting the addition of desmopressin to the treatment regimen, along with ibuprofen, hydrochlorothiazide–amiloride, and sildenafil. However, due to a continued lack of clinical improvement, the patient was referred to a high complexity reference hospital for multidisciplinary management.

**Figure 1:**
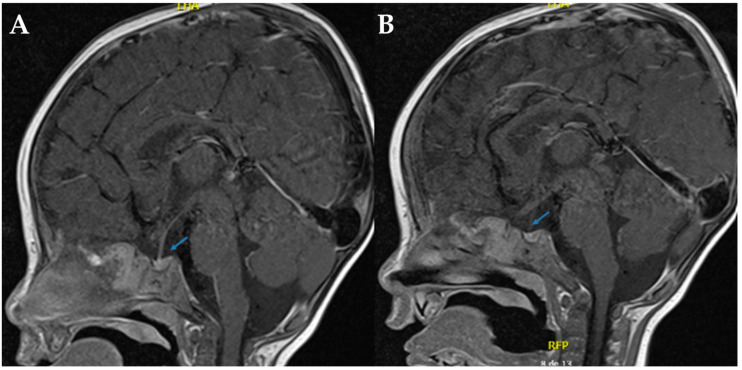
Sella turcica in T1 sagittal brain MRI. (**A**,**B**). In both sections, the arrows indicate the absence of the hyperintense signal characteristic of the neurohypophysis, which is compatible with its radiological absence.

Magnetic resonance imaging (MRI) of the sella turcica revealed the absence of the posterior pituitary bright spot, reinforcing the suspicion of mixed diabetes insipidus (See Figure 1). Despite continued desmopressin administration, the patient exhibited no improvement in polyuria or hypernatremia. After a comprehensive evaluation by pediatric nephrology and endocrinology, it was concluded that the radiological findings did not confirm concomitant CDI. Consequently, desmopressin therapy was discontinued.

A final diagnosis of NDI was confirmed, and the patient’s management was adjusted to include hydrochlorothiazide, ibuprofen, exclusive breastfeeding, and a high-calorie, low-sodium, and low-protein diet. With progressive clinical improvement, the patient was discharged with outpatient follow-up by pediatric nephrology.

**Figure 2:**
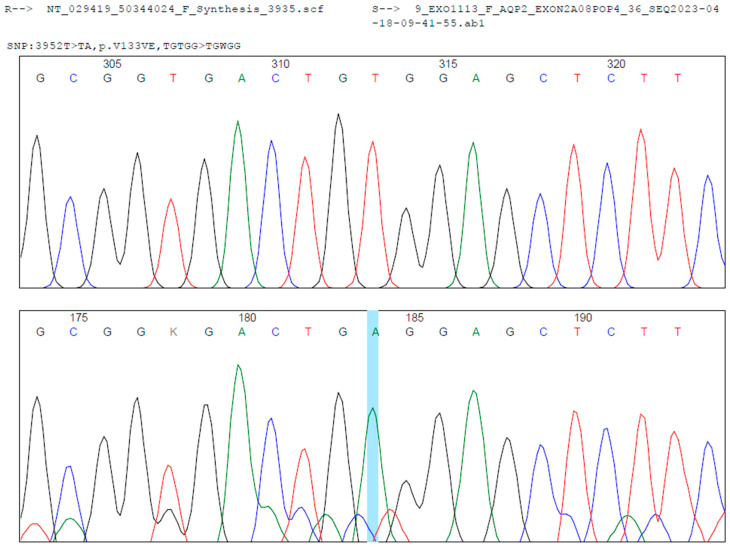
Sanger sequencing analysis shows a homozygous c.398T > A transition in the exon 2 of the *AQP2* gene (Blue Line, middle). In the upper localization is the reference sequence. Below is the case sequence. Mutation Surveyor^®^ was used for the bioinformatic analysis.

During hospitalization, the patient underwent clinical genetics evaluation and targeted massively parallel sequencing (next-generation sequencing, NGS) for diabetes insipidus-related genes (*AQP2*, *AVP*, and *AVPR2*) at the Department of Pathology and Laboratory Medicine of FVL. A novel homozygous missense variant, NM_000486.6:c.398T > A (p.Val133Glu), was identified in exon 2 of the *AQP2* gene. According to the American College of Medical Genetics and Genomics (ACMG) criteria [1]—PM2 (absent from population databases), PP2 (missense variant in a gene with low benign variation), and PP3 (computational evidence suggesting a deleterious effect)—and following review by the FVL Medical Genetics Board, this variant was classified as likely pathogenic. It is consistent with the patient’s clinical phenotype of NDI. The variant has not been previously reported in the Human Gene Mutation Database (HGMD), NCBI ClinVar, gnomAD, or the 1000 Genomes Project. Sequencing Sanger confirmation was performed (see Figure 2). Segregation analysis in the parents could not be performed due to loss to follow-up for administrative and social reasons.

## 3. Literature Review and Discussion

AVP is a hormone secreted by the neurohypophysis that plays a pivotal role in water homeostasis. It exerts its action by binding to the vasopressin V2 receptor (AVPR2) on the basolateral membrane of principal cells in the renal collecting duct, promoting the insertion of aquaporin-2 (AQP2) channels into the apical membrane and thereby increasing water reabsorption. AQP2 is the primary AVP-regulated water channel located in the collecting duct and distal convoluted tubule [2,4].

AVP secretion is tightly controlled by two main feedback mechanisms: osmoregulation, which responds to changes in plasma osmolality as small as <1%, and baroregulation, which responds to 5–10% changes in blood volume and ≥5% changes in mean arterial pressure [4]. AVP regulation of AQP2 may occur over both short- and long-term timescales. In the short term, AVP induces trafficking of AQP2 from intracellular vesicles to the apical membrane. Prolonged AVP stimulation (≥24 h) results in increased transcription and expression of AQP2, amplifying the renal capacity to reabsorb water [2,6]. In addition, AVP enhances the permeability of the collecting duct to sodium and urea by regulating transporters such as the urea transporter A1 (UT-A1), further concentrating the urine. In the absence of AVP action—as occurs in NDI—the collecting duct remains impermeable to water, sodium, and urea, markedly impairing water reabsorption and predisposing the patient to dehydration and hypernatremia [2,6].

Under normal physiologic conditions, AVP can elevate urine osmolality up to 1200 mOsm/Kg and decrease urine output to approximately 700–800 mL/day. When plasma osmolality normalizes, AVP levels decline, and AQP2 expression on the apical membrane is downregulated to restore the water balance [4].

Diabetes insipidus (DI) is part of the polyuria-polydipsia syndrome, which is defined by a urine output exceeding 50 mL/Kg/day of hypotonic urine (<300 mOsm/Kg H_2_O), accompanied by polydipsia and polyuria [3,7,10]. Within the differential diagnosis of this syndrome are NDI, central diabetes insipidus (CDI), and primary polydipsia—including both dipsogenic and psychogenic forms [3,4]. Distinguishing among these entities is essential, as treatment strategies differ significantly, and inappropriate management may lead to serious consequences [3].

NDI, also referred to as arginine vasopressin resistance (AVP-R) [5], is a rare disorder in which ADH or AVP is produced normally but fails to elicit a response in the renal collecting ducts. This renders the kidneys unable to concentrate urine, despite elevated AVP levels [2,3]. The cardinal clinical manifestations of NDI include polyuria—with thresholds defined as >150 mL/Kg/day in neonates, 100–110 mL/Kg/day in infants ≤ 2 years of age, and 40–50 mL/Kg/day in children > 2 years of age—along with hyposthenuria and compensatory polydipsia [2,7,11,12,13]. When these features are caused by impaired AVP synthesis or secretion, the diagnosis is CDI, also known as arginine vasopressin deficiency (AVP-D), which is the most common form of DI overall [5]. Still, in the pediatric population, NDI is more prevalent than CDI [7]. Both NDI and CDI can be either acquired or hereditary, with acquired forms being more frequent [3,4,6].

The diagnostic workup for polyuria-polydipsia syndrome includes measurement of plasma osmolality. A value > 295 mOsm/Kg supports a diagnosis of DI, while values < 285 mOsm/Kg suggest primary polydipsia [4]. Urine osmolality in both CDI and NDI typically remains < 200–300 mOsm/Kg [2,4,7]. Characteristic laboratory findings in NDI include urine output > 4 mL/Kg/h, serum sodium > 170 mmol/L, serum osmolality > 300 mOsm/Kg, urine osmolality < 300 mOsm/Kg, and urine specific gravity < 1.005 g/mL [7,8]. Our patient fulfilled all these criteria for clinical diagnosis.

The overall prevalence of DI is estimated at 1 in 25,000 individuals, or approximately 0.004% of the global population. It has no sex predilection and may present at any age, although the hereditary forms tend to manifest earlier in life [4]. Hereditary NDI affects an estimated 5 in every 5 million males and is caused by loss-of-function mutations in either the *AVPR2* gene (X-linked; 90–98% of cases, with an incidence of 4–8 per million live births) or the *AQP2* gene (autosomal recessive, 2–10%; autosomal dominant, ~1%) [2,4,6,7,8]. A significant disruption in AVP signaling generally requires the inactivation of both alleles, explaining the predominance of autosomal recessive inheritance in *AQP2*-related NDI [2].

Approximately 4% of clinically diagnosed NDI cases yield negative genetic testing results. In these instances, the presumed etiology is either an intronic variant in *AVPR2* or *AQP2*, or a pathogenic variant in a yet-undiscovered gene involved in the AVP-AQP signaling pathway [8]. No definitive genotype–phenotype correlation has been established for NDI, and mutations in both *AVPR2* and *AQP2* may lead to either complete or partial forms of the disease [8,14]. Nonetheless, autosomal dominant *AQP2* mutations tend to present with milder symptoms and later onset compared to autosomal recessive variants [10]. This observation aligns with our patient’s clinical course, as he presented with early and severe symptoms of NDI secondary to a homozygous missense variant in exon 2 of *AQP2* (c.398T > A; p.Val133Glu). Previous compound heterozygous and homozygous cases in *AQP2* have shown higher hypernatremia and serum osmolality values and lower urine osmolality than non-compound heterozygous cases [15]. These cases included two with one mutation (p.G100R; p.G165D) near our case mutation (p.V133E), which were also located in a transmembrane domain (III and V) [15]. Clinical presentations did not differ significantly according to the mutation reported [15]. Another similar case (p.L137P) reported a worse clinical disease [16], but in another (p.A130V), clinical data was not available [17].

Our variant affects a moderately conserved region of the gene, replacing valine (a nonpolar amino acid) with glutamic acid (a polar, negatively charged amino acid), which could potentially alter protein folding and function, as in silico predictive algorithms uniformly indicate a deleterious impact on protein function. Furthermore, the absence of this variant from major population databases strengthens its pathogenicity. According to ACMG criteria, it meets criteria for a “likely pathogenic” classification [1]. Missense mutations cause a misfolded protein with its retention in the endoplasmic reticulum and no translocation to the apical membrane [18,19]. Currently, all *AQP2* mutations causing recessive NDI are located throughout the six transmembrane domains and five connecting loops of AQP2 [6]. As our mutation is in the IV transmembrane domain, a deleterious effect on folding and anchoring to the apical membrane can be predicted. Homozygous mutations cause a non-functional protein that is retained in the endoplasmic reticulum as evidenced by their scattered ‘reticular’ expression pattern, by their unglycosylated or high mannose glycosylated variants, and by their decreased stability in comparison with wild-type AQP2 [6]. Consistent with the recessive nature of inheritance and possibly because of their misfolding, these AQP2 mutants exist as monomers that are incapable of heteroligomerizing with and impeding the routing and maturation of wild-type AQP2 in its pathway to the apical membrane [6].

To date, over 300 mutations in *AVPR2* have been identified, most of which are missense mutations (62%) or small deletions (18%). In contrast, approximately 70 disease-causing variants have been reported in *AQP2*, with nonsense mutations accounting for 77% [6,7,8].

Early diagnosis is crucial to prevent life-threatening episodes of dehydration and hypernatremia (sodium > 145 mEq/L), as well as progressive kidney damage due to urinary tract dilation and obstructive nephropathy [2,7]. Most diagnoses of hereditary NDI occur during the first year of life, with a median age at diagnosis of 0.6 years [8], consistent with our case in which diagnosis and treatment were initiated within this critical window.

Familial cases of NDI due to X-linked *AVPR2* mutations can be traced by identifying affected males and heterozygous female carriers [2]. Although often asymptomatic, heterozygous females—who represent 1–25% of X-linked NDI cases—may exhibit variable clinical phenotypes, including severe disease, due to skewed X-chromosome inactivation favoring the mutant allele [2,8].

Male patients with *AVPR2* mutations typically present with severe symptoms during the neonatal period, including early-onset hypernatremia, reduced sodium excretion, and inappropriately low urine osmolality [2,7]. Clinical signs often emerge within the first week of life and may include irritability, diarrhea, nausea, and frequent vomiting despite adequate attempts at breastfeeding, unless water is offered first [2,7,8]. Persistent symptoms can include poor feeding, failure to thrive, dehydration (evidenced by depressed fontanelle, decreased skin turgor, or enophthalmos), and constipation. Other less specific manifestations include intermittent fever, nocturia, enuresis, postural hypotension, and developmental delay [2,7,8].

If undiagnosed or untreated, children may suffer from recurrent hypernatremic dehydration, which can result in seizures, intellectual disability, and even mortality [2,7,8]. Moreover, chronic excessive water intake and compensatory restriction of salt and protein intake may result in hypocaloric dwarfism [2]. High urinary volumes increase the risk of obstructive uropathy, including megacystis, hydronephrosis, hydroureter, and recurrent urinary tract infections [2,7,8]. Severe dehydration episodes may lead to hypoperfusion and long-term neurodevelopmental impairment, which were historically common before early diagnostic tools and treatment options became available [2].

Notably, the clinical severity of NDI caused by *AQP2* mutations is comparable to that observed in *AVPR2*-related disease [2]. The female carriers—either heterozygous or homozygous for *AQP2* mutations—can display phenotypes as severe as those seen in *AVPR2* heterozygous carriers [2]. Our case was born from non-consanguineous parents, but a variant in homozygosis was detected, which could likely be related to a founder effect in the origin zone of the patient due to a higher risk of inbreeding/endogamy, well known in this zone.

The initial diagnostic workup for suspected DI includes a thorough clinical history and confirmation of polyuria via a 24 h urine collection [7]. Additional evaluations should include measurements of plasma osmolality, baseline urine osmolality and specific gravity, and serum levels of electrolytes (sodium, potassium, and calcium), glucose, blood urea nitrogen (BUN), and creatinine. These parameters establish a baseline for monitoring disease progression and treatment response [4,7].

The water deprivation test, which involves prolonged fasting from fluids (typically ≥17 h) to induce a rise in plasma osmolality > 150 mOsm/Kg or a 3–5% reduction in body weight, followed by the administration of exogenous AVP (desmopressin), is traditionally used to assess renal concentrating ability and diagnose diabetes insipidus (DI) [2,3,4]. A <50% increase in urine osmolality following desmopressin administration is considered diagnostic for NDI [4]. In healthy individuals, urine osmolality typically exceeds 800 mOsm/Kg after water deprivation and shows little or no further increase with desmopressin. If urine osmolality exceeds 1000 mOsm/Kg in a single sample or >600 mOsm/Kg in two consecutive samples, DI is effectively excluded, and the test should be terminated [4,11]. Conversely, in both central diabetes insipidus (CDI) and NDI, urine osmolality remains <200–300 mOsm/Kg [4,10].

Despite its long-standing use as the diagnostic gold standard for distinguishing among AVP resistance (NDI), AVP deficiency (CDI), and primary polydipsia, the water deprivation test has limitations. Its diagnostic accuracy ranges from 40% to 70%, and the procedure is both labor-intensive and challenging, particularly in infants and young children [2,7]. In addition, measuring circulating AVP is technically difficult due to its instability and lack of reliable commercial assays [2].

Measuring AVP levels directly or indirectly can help differentiate between renal resistance to AVP (NDI) and impaired AVP secretion (CDI) [2]. However, in neonates, infants, or any patient with hypernatremia or hyposthenuria, water deprivation testing is contraindicated due to significant safety concerns [7,8]. In our case, a water deprivation test was not performed due to the patient’s age and safety. In such cases, a combination of serum osmolality > 300 mOsm/Kg and urine osmolality < 600 mOsm/Kg is sufficient to support a diagnosis of DI.

The desmopressin challenge test offers a safer and more practical alternative in these scenarios. Intranasal administration (10 µg for children < 1 year; 20 µg for those >1 year) or intravenous administration (0.5 µg/m^2^ body surface area in children; 2 µg in adults), followed by urine collection over the next 4–5.5 h (or 2 h post-IV administration), allows for the differentiation of NDI. A <50% increase in urine osmolality confirms NDI, with an increase < 10% suggesting complete AVP resistance [8]. In our case, the desmopressin challenge test performed on two separate occasions failed to elicit a meaningful increase in urine osmolality, confirming the diagnosis of NDI.

Recently, the measurement of copeptin—a stable peptide derived from the AVP precursor and secreted in equimolar amounts—has emerged as a promising biomarker for differentiating polyuria-polydipsia syndromes [3,4]. Copeptin levels can be measured within 2 h using only 50 μL of plasma or serum [4]. A copeptin level ≥ 21.4 pmol/L (without prior stimulation) is highly specific for NDI [3,4]. Under osmotic stimulation (e.g., hypertonic saline infusion), a copeptin cutoff of 4.9 pmol/L differentiates CDI from primary polydipsia with a diagnostic accuracy of 96–97% (95% for partial CDI) [3,4]. When using non-osmotic stimulation with arginine, a cutoff of 3.8 pmol/L achieves 93% accuracy (90% for partial CDI) [3]. This test is not available in our setting.

Early genetic testing is strongly recommended for neonates or young children presenting with signs suggestive of NDI or unexplained dehydration [8]. The identification of a hemizygous or heterozygous pathogenic variant in *AVPR2* or a homozygous or heterozygous variant in *AQP2* confirms the diagnosis and has significant implications for prognosis and clinical management [8]. Genetic testing is also helpful in identifying partial NDI cases where conventional testing may be inconclusive [8]. A targeted multigene panel using massively parallel sequencing—including at minimum *AQP2*, *AVPR2*, and, when CDI is considered, *AVP*—is recommended to improve diagnostic yield while limiting incidental findings [8,10]. Genetic counseling is essential for affected individuals, asymptomatic carriers, and family members at risk. Counseling should address inheritance patterns, reproductive options, and potential risks to future offspring [8].

In our case, molecular diagnosis was not delayed, and a targeted exome sequencing for DI revealed a novel, homozygous missense variant—classified as likely pathogenic—in the *AQP2* gene. Due to administrative and social constraints related to follow-up, segregation analysis in the parents could not be performed. Although both parents were asymptomatic and showed no clinical signs of NDI at the time of evaluation, they are presumed to be carriers of the identified mutation due to the higher risk of consanguinity in their region of origin.

Initially, CDI was suspected due to the absence of a visible neurohypophysis on neuroimaging. However, the lack of clinical or biochemical response to desmopressin administration, combined with the confirmatory genetic test, ultimately established the diagnosis of NDI. This has been reported previously in other cases with NDI [20,21] and is possibly related to chronic dehydration with subsequent depletion of pituitary AVP, proposing that the bright signal in neurohypophysis reflects neurosecretory granules containing vasopressin, and this signal depends on the production and secretion of AVP [21]. Nevertheless, idiopathic absence of this hyperintensity has also been proposed [21] and can even be physiologically absent in ~10% of healthy individuals [21]. Therefore, MRI does not necessarily reflect the origin of DI (either CDI or NDI) and should only be performed in diagnosed cases of CDI [21]. This highlights the critical value of molecular testing in cases with inconclusive clinical findings or overlapping phenotypes.

All complications associated with congenital NDI are preventable with adequate hydration [2]. From birth, unrestricted access to water should be ensured [2]. In cases of hypernatremia, standard management includes an intravenous infusion of 5% dextrose in water or hypotonic saline, aiming to reduce serum sodium levels by 0.5 mEq/L per hour [7]. Infants should be offered fluids every two hours, and supplemental enteral feeding via nasogastric or gastrostomy tube may be necessary during the night [8]. If IV fluids are indicated, hypotonic solutions (0.22–0.25% saline) are preferred to avoid worsening hypernatremia [8].

Nutritional support involves a low-sodium, low-protein (2 g/Kg/day), and low-phosphorus diet. Pharmacological management includes thiazide diuretics (hydrochlorothiazide 1–4 mg/Kg/day), often combined with amiloride (0.3–0.6 mg/Kg/day) to mitigate hypokalemia, or alternatively, indomethacin (0.75–2 mg/Kg/day). Ibuprofen (20–25 mg/Kg/day) may be used where indomethacin is unavailable [2,7,8]. These interventions can reduce urine output by up to 70% [8]. In cases of growth retardation, protein intake may be increased to 3 g/Kg/day, but sodium intake should remain significantly restricted (<1 mEq/Kg/day or ≤100 mEq/day; in severe cases, as low as 9 mEq/day) [7,10]. Breast milk or low-solute infant formulas are preferred in early infancy [7,8].

Potential renal solute load (PRSL) plays a key role in fluid management. PRSL may be calculated using formulas proposed by Ziegler and Fomon or Nevin-Folino and Miller [7]. A low-solute diet should always be maintained alongside pharmacologic treatment to control symptoms [2,4,7]. An osmotic load in dietary intake of 15 mOsm/Kg/day is recommended for a child with NDI and patients with significant renal impairment [8]. The diet was adjusted in our case to meet this requirement.

When conventional therapies are insufficient or unavailable, experimental treatments such as metformin, sildenafil, clopidogrel, simvastatin, sodium nitroprusside, or prostaglandin receptor agonists have been explored [7,8,22]. AVPR2 antagonists (both selective and non-selective) have shown the potential to restore AVPR2 localization to the plasma membrane, improving urine concentration, although further research is needed to confirm efficacy [8,22].

In our case, hydrochlorothiazide (5.5–6 mg every 8 h), ibuprofen (50–60 mg every 6–12 h), exclusive breastfeeding on demand, and a high-calorie, low-sodium, and low-protein diet (150 mL maternal milk plus 15 g Nessucar-maltodextrin-NESTLE^®^ and with each food, no milk formula, protein in soup) resulted in clinical improvement. Desmopressin (10–120 μg every 24 h) was suspended due to a lack of response and the exclusion of CDI. Other therapies offered during hospitalization were hydrochlorothiazide-amiloride and sildenafil (the doses are not available, as they were administered before hospital referral). The therapy was titrated according to the patient’s clinical response and coupled with dietary adjustments. Adequate post-discharge hospital follow-up was not performed due to administrative barriers. During a pediatric nephrology ambulatory evaluation, the patient was successfully following the diet (only breastfeeding 5–6 ounces per day plus Nessucar 10 g per 3 ounces), serum sodium levels were normal, and diuresis had levelled off.

The paradoxical antidiuretic effect of thiazide diuretics results from natriuresis-induced volume contraction, leading to decreased GFR and increased sodium and water reabsorption in the proximal tubules—possibly augmented by carbonic anhydrase inhibition [4,8]. Similarly, nonsteroidal anti-inflammatory drugs (NSAIDs) reduce GFR by inhibiting COX-2–mediated vasodilation of the afferent arteriole, thereby limiting sodium and water excretion [8]. Chronic NSAID use carries a risk of acute kidney injury; however, this risk is not increased in patients with NDI. In this case, NSAID therapy was initiated after careful consideration of the risk–benefit ratio for the patient. Other investigational therapies, including secretin, fluvastatin, and other statins, remain under active study [4].

Importantly, isotonic saline infusion is contraindicated in NDI, as it may exacerbate hypernatremia due to the renal water loss and sodium retention inherent to the disorder [4].

As illustrated in our case, the management of NDI requires a multidisciplinary team, including a pediatric endocrinologist, nephrologist, geneticist, pediatrician, and nutritionist, to achieve optimal outcomes [7]. In this patient, a comprehensive approach—combining pharmacologic treatment, dietary adjustments, and coordinated care—resulted in a favorable clinical course and hospital discharge with plans for multidisciplinary outpatient follow-up. Unfortunately, such follow-up could not be conducted at our institution due to administrative constraints, but it is currently being provided in the patient’s city of origin.

## 4. Conclusions

The diagnosis of nephrogenic diabetes insipidus (NDI) within the context of the polydipsia-polyuria syndrome requires a multidisciplinary approach and the integration of advanced diagnostic tools, including molecular genetic testing. In this case, the identification of a novel, likely pathogenic, homozygous missense variant in *AQP2* (c.398T > A; p.Val133Glu) was associated with early-onset and severe clinical manifestations. This highlights the importance of molecular diagnosis in complex or atypical presentations.

## 5. Limitations

Segregation analysis of the *AQP2* variant in the patient’s parents could not be performed due to administrative and social constraints related to follow-up, representing a limitation in confirming familial carrier status.

## Figures and Tables

**Table 1:** Clinical and laboratory data.

	Description
Signs/symptoms	Thrive failure, generalised pallor, polyuria, polydipsia, irritability-crying, severe dehydration, and systolic murmur
Urinary output (mL/Kg/h)	5.9
Serum sodium (mmol/L)	158
Serum chloride (mmol/L)	121
Urinary density (g/mL)	1004
Urinary osmolarity (mosm/Kg H_2_O)	42
Serum osmolarity (mosm/Kg H_2_O)	320

## Data Availability

The original contributions presented in this study are included in the article. Further inquiries can be directed to the corresponding author.
